# Obstacles to emergency medical consultation in cases of conflict-related sexual violence

**DOI:** 10.1371/journal.pone.0317082

**Published:** 2025-01-28

**Authors:** Denis Mukwege, Gilbert M. Mugisho, Raha Maroyi

**Affiliations:** 1 Panzi General Referral Hospital, Bukavu, The Democratic Republic of Congo; 2 School of Medicine, Université Evangélique en Afrique (UEA), Bukavu, The Democratic Republic of Congo; 3 International Center for Advanced Research and Training (ICART), Bukavu, The Democratic Republic of Congo; 4 Faculty of Economics, Université Evangélique en Afrique (UEA), Bukavu, The Democratic Republic of Congo; Sefako Makgatho Health Sciences University, SOUTH AFRICA

## Abstract

**Background:**

Despite the availability of a well-developed holistic care model for victims of conflict-related sexual violence, little is known about the factors that determine late presentation for care post-sexual violence care. Drawing from data from the Democratic Republic of the Congo, this study aimed to determine obstacles to accessing emergency medical care within 72-hours of sexual violence (SV).

**Methods:**

We retrospectively analyzed data from 4048 victims of SV treated at Panzi Hospital (PH) in Bukavu city between 2015 and 2018. The factors of access to care within 72h were analyzed using logistic regression.

**Results:**

88% of the victims consulted after 72h post sexual violence. Several sociodemographic factors were found to limit access to the medical care post-sexual violence including the victim’s age (p = 0,022), place of residence (p = 0,000) and education level (p = 0,039). Clinical discomfort from pain during urination (p = 0,002) and fear of pregnancy (p = 0,000) were also associated with late assessment of care.

**Conclusion:**

Seeking medical care within 72 hours after sexual violence within the critical 72-hours timeframe is crucial to avoid several medical complications stemming from SV. Improvement will be achieved by integrating the post-exposure prophylaxis protocol into primary health care, as well as by increasing community awareness of the relevance of timely consultation after sexual abuse.

## Background

Sexual violence (SV) is defined by the World Health Organization (WHO) as “any sexual act, attempt to obtain a sexual act, unwanted sexual comments or advances, or acts to traffic, or otherwise directed, against a person’s sexuality using coercion, by any person regardless of their relationship to the victim, in any setting, including but not limited to home and work.” [1] SV is considered a health emergency, and those experiencing this situation often suffer from multiple traumas, including physical, emotional, mental, and social consequences [2–5]. Therefore, early prophylactic treatment is warranted to minimize these deleterious effects [2,4]. The goals of medical care are multifactorial and include prevention of unwanted pregnancies, sexually transmitted diseases, HIV, and psychological disorders. Further care may be required for related physical traumas, including bruises, wounds, and traumatic genital fistulas, in cases of violent rape [2–4].

While SV may occur in any context, it is often used in conflict settings, targeting women and girls, and as such, has been reported as a weapon of war [6]. The perpetrator’s action consequently extends to the family unit with the intention of destroying it [7–11]. During the war time since 1996, rape was systematic and committed against both adults and children, often resulting in the transmission of sexually transmitted infections (STIs), including human immunodeficiency virus-acquired immunodeficiency syndrome (HIV/AIDS), and complex injuries reflecting the brutality of violence [6]. In 2011, in DRC, the term ‘rape with extreme violence’ was described as characterized by (i) gang rape, usually by three or more perpetrators, with high risk of injury; (ii) genital mutilation; and (iii) intentional transmission of STIs such as chlamydia and HIV/AIDS [12].

The social fabric is destroyed by public rape and torture, resulting in a loss of community identity and social cohesion. Unintended pregnancies due to rape contribute to further psychological trauma and socioeconomic hardships in families. All these factors lead to community destabilization; indigenous communities are often displaced, and these territories become occupied by perpetrators [13].

Despite increasing attention to sexual violence in conflict, many studies still struggle to fully document or explore the many complex factors that influence survivors’ decisions to seek care [14,15].

In this context, Panzi Hospital (PH) has developed an innovative and holistic care model to provide the medical, psychosocial, legal, and socioeconomic needs of survivors of SV [1]. However, medical consultation is often delayed, occurring after the recommended 72-hour timeframe, particularly for the care of victims of sexual violence [16,17], This hinders preventive efforts for both psychological disorders and medical complications, including pregnancy prevention and reduction of STI transmission, for which medications must be administered within 72-hours of the SV incident. This also affects the documentation and care of physical injuries required to enable legal action [2].

This study, conducted at PH, aimed to determine obstacles to medical care within 72 hours following an SV encounter in the Eastern DRC. The outcomes of this study will inform effective and efficient preventative policy strategies to improve the quality of life of victims of SV.

## Methods

### Type of study

This retrospective cohort study used data collected from 4048 sexual assaulted victims between 2015 and 2018. The present study was approved by The National Health Ethics Committee, DRC (CNES 001/DPSK/200PP/2022).

### Study framework

This study was conducted in a clinic for victims of SV at PH in the Eastern DRC. PH was established in 1999 in the wake of the first and second Congo wars in response to the breakdown of health infrastructure that resulted from ongoing conflict, coupled with reports of mass rape. PH now serves as a center of excellence for the care of SV victims in women and girls [2]. PH has developed a holistic “one-stop-shop” model of care for SV survivors that consists of four pillars, accounting for medical, psychosocial, legal, and socioeconomic needs [2,18]. Between 1999 and 2019, 56,050 victims of SV benefited from this holistic care model. Ultimately, personalized care delivered by PH had a positive community impact [2].

### Study population

We included all victims without age restrictions admitted within and after a 72-hour time frame during the study period. Our sample consisted of 4048 victim files that we accessed during data collection.

### Variables, data source and collection

All the victims provided written informed consent to obtain data from their medical records. The data used in this study were extracted from a fully anonymized electronic database of victims of SV at PH in January 2023.

The study variables included sociodemographic characteristics such as age (< 18 years and ≥18 years), place of residence (Bukavu city or urban = 0, outside Bukavu or rural = 1), marital status (married: yes/no), education level (none, primary, secondary, or more), facilitative care seeking (yes = by a health facility, police, local civilian society; no = coming alone), information on SV (torture, history of rape, number of aggressors, type of aggressor, relationship to the perpetrator), and socioeconomic vulnerability (no = ≥50 USD; yes = < 50 USD per month).

We were interested in the circumstances of SV and determined the following parameters: physical torture (no, yes), previous rape (no, yes), number of perpetrators (one, two, or more), relationship with the perpetrator (no, yes), and type of perpetrator (armed, civilian).

Clinical information, including genital irritation (no, yes), vaginal bleeding (no, yes), leucorrhea (no, yes), sensation of a foreign body in the vagina (no, yes), Fear of Pregnancy (no, yes), and painful urination (no, yes), was also collected. Information regarding pregnancy was age dependent.

The dependent variable was access to post-sexual violence medical care within the critical 72-hour period [18]. This variable was defined as follows: within 72 and 72 hours. Timely access to care was considered if the victim arrived at the PH within 72 hours following the assault.

### Data management and analysis

The collected data were exported from Epi Info to Excel for cleaning. Data analysis was performed using Statistical Package for the Social Sciences (SPPS 20; IBM, Armonk, NY, USA). Categorical variables were presented as percentages and frequencies; ordinal variables were presented as medians and ranges. Cross-tabulation and Chi-square analysis allowed for a dependency link between the explained variable (consultation within 72 h) and the explanatory variables. A binary logistic model was used to analyze the factors associated with access to care within 72 h of PH. Threshold 0.05 was used. The significance of the coefficients was tested using the Wald’s test.

## Results

**Fig 1:**
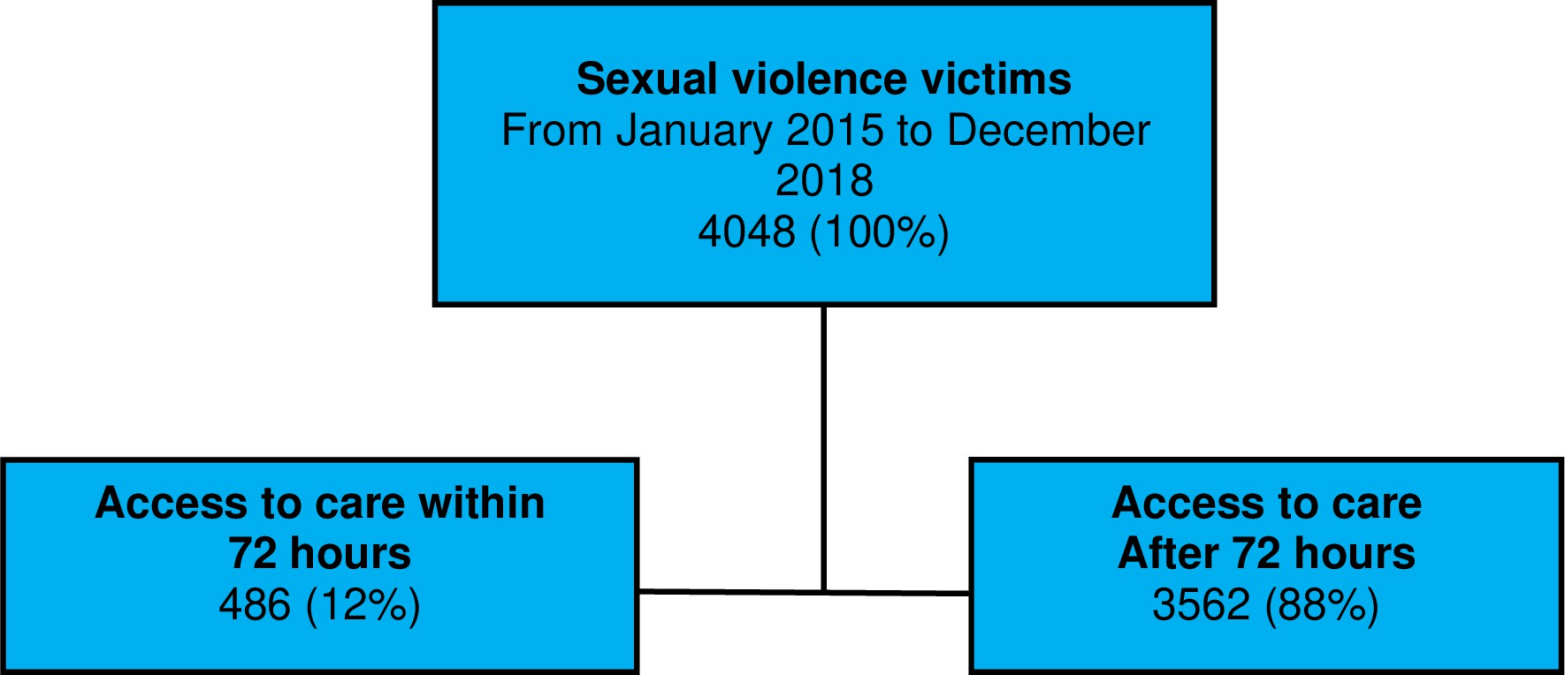
Distribution of sexual violence victims and medical consultation within the critical 72-hour timeframe in the holistic care model.

During the study period, we found that 488 (12%) of the victims accessed medical services within 72 hours after SV, whereas the majority (3562; 88%) consulted after 72 hours (Fig 1).

### Socio-demographic parameters

Sociodemographic data showed that among all SV consulted within 72 hours, 282 (58%) were underage compared to adults 204 (42%). We found that 374 (77%) were unmarried compared to married people 112 (23%). Additionally, 271 SV (56%) admitted within 72 hours were coming from Bukavu city compared to those from rural area 212 (44%).

Timely access to healthcare services also depends on educational level. Victims with primary and secondary education levels were likely to come to the hospital within a frame time limit of 72 hours: 169 (35%) and 166 (34%), respectively. We found that 343 (71%) of SV admitted within the 72-hour window were referred by partner organizations to PH, compared to 143 (29%) came on their own within the 72h window. Among victims with higher income 422(87%) consulted within the acceptable time frame compared to 64(13%) in the vulnerable condition (Table 1).

**Table 1:** Socio-demographic information on survivors of sexual violence.

Variables	Categories	After 72 hours	Within 72 hours
		n (%)	n (%)
Age (years)	< 18	930 (24)	282(58)
Mean : 30.3 years	> 18	2632(74)	204(42)
Provenance	Bukavu city (Urban)	628 (18)	271(56)
	Outside Bukavu (Rural)	2934(82)	215(44)
Marital status	Married	1726(48)	112(23)
	Other	1836(52)	374(77)
Education level	None	1689(47)	151(31)
	Primary	1102(31)	169(35)
	Secondary and more	771 (22)	166(34)
Facilitated care seeking	No	495 (14)	143(29)
	Yes	3067(86)	343(71)
Socio-economic vulnerability	No	2985 (84)	422(87)
	Yes	577 (16)	64 (13)

### SV circumstances and perpetrator profiles

The circumstances surrounding the rape revealed that 59 (12%) victims were tortured during the SV consultation within 72 h. There was a similar percentage (12%) of victims with a history of rape and no history of sexual assault in terms of seeking medical services.

When comparing the number of perpetrators, 361 (19%) victims were abused by a single perpetrator and arrived at the hospital within 72 hours. We noted that 430 (89%) of those sexually abused by civilians and 212 (44%) with relationships with perpetrators came in the timeframe to the hospital for treatment (Table 2).

**Table 2:** Circumstances of sexual violence and profile of the perpetrator.

Variables	Categories	After 72 hours	Within 72 hours
		n (%)	n (%)
Physical torture	No	3154(88)	427(88)
	Yes	408 (87)	59 (12)
Previous rape history	No	3133(88)	427(88)
	Yes	429 (88)	59 (12)
Number of perpetrators	One	1568(81)	361(74)
	Two and more	1994(94)	125(26)
Type of perpetrator	Armed	1081(95)	56 (11)
	Civilian	2481(85)	430(89)
Relationship with the perpetrator	No	2668(91)	274(56)
	Yes	894 (81)	212(44)

### Health status of the victim

We found that the victims who came within the deadline presented some clinical signs, notably genital irritation 54(58%), vaginal bleeding 6(7%), vaginal sensation of a foreign body 5(6%), leucorrhea 48(55%).

In addition, victims with painful urination 161(62%) and fear of pregnancy 12(5%) consulted within the required timeframe (Table 3).

**Table 3:** Clinical related information after sexual violence and access to medical care.

Variables	Categories	After 72 hours	Within 72 hours
		n (%)	n (%)
Genital irritation	No	206(24)	39(42)
	Yes	639(76)	54(58)
	Total	845(100)	93(100)
Vaginal bleeding	No	768(94)	83(93)
	Yes	49(6)	6(7)
	Total	817 (100)	89(100)
Sensation of foreign body in the vagina	No	623(77)	81(94)
	Yes	183(23)	5(6)
	Total	806(100)	86(100)
Leucorrhoea	No	184(22)	40(45)
	Yes	638(78)	48(55)
	Total	822(100)	88(100)
Fear of pregnancy	No	295(71)	206(95)
	Yes	119(29)	12 (5)
	Total	414(100)	218(100)
Painful urination	No	708(25)	100(38)
	Yes	2162(75)	161(62)
	Total	2870(100)	261(100)

### Factors associated with seeking care within 72 h post-rape

The results of the multiple logistic regression (Table 4) enabled us to understand the factors associated with care seeking within 72 h. Considering the thresholds of 1 and 5%, respectively, we found that the victim’s origin, age, level of education, and painful urination were factors associated with seeking medical attention within 72 hours of exposure.

**Table 4:** Multiple regression of associated factors on seeking care within 72 hours post rape.

Variables	Estimations (β)	p-value	OR	Confidence intervals Exp(β) 95%	
				Lower limit	Upper limit
**Age**	0.707	.022	2.027	1.106	3.715
**Origine**	-1.112	.000	.329	.198	.547
**Marital status**	0.400	.255	1.493	.749	2.974
**Education**	-0.625	.039	.535	.296	.969
**Perpertrator type**	0.324	.414	1.383	.635	3.013
**Fear of Pregnancy**	-1.889	.000	.151	.071	.324
**Painful urination**	-0.701	.002	.496	.320	.769

OR = Odds ratio, Exp (β) = expected estimations.

## Discussion

The results of this study indicated a high rate of delay in seeking emergency medical care for conflict-related sexual violence. Various obstacles were identified, including victims over 18 years of age, those form rural areas, with lower levels of education, experiencing uncomfortable symptoms such as painful urination, and concerns about unwanted pregnancies.

Although it is the ideal and right of the victim, timely access to emergency medical care remains a major problem. Victims of conflict-related sexual violence have particular hurdles in reporting and receiving timely care when compared to survivors of other types of gender-based violence [19]. While both conflict and non-conflict settings present barriers to accessing care following sexual violence, the nature and severity of the barriers differ significantly. In conflict-affected environments, existing vulnerabilities are often intensified, resulting in a more complex interplay of fear, stigma, and disruption of services. In contrast, although non-conflict settings also pose challenges, they generally provide a more stable context in which survivors may have greater opportunities to seek assistance [20].

Our findings revealed that some hurdles to care, such as the victim’s age, contributed to this delay. In contrast to previous studies [21,22], our results showed that individuals under 18 years of age were more likely to access care within 72 hours than adults. Some reasons, such as children’s social dependence [18] and the responsibility of parents or guardians have facilitated timely access to quality post-violence care [23].

Studies have shown that parents of children who have experienced sexual abuse often report high levels of guilt, shame, and anger regarding their child’s abuse [24]. These emotional responses can complicate the parent-child relationship and may affect the parent’s ability to provide adequate support and care to the child [25]. Furthermore, community support and attitudes regarding child sexual abuse might influence reporting rates and efficiency of support services, as demonstrated by Abeid et al. [26] in Tanzania.

The issue of prompt access to emergency care for victims, especially quality care, is well documented and remains an issue in the ongoing conflict in the Democratic Republic of the Congo.

In the present study, the rate of delayed access to care was 88%. Several studies have reported rates of delay in less than 60% cases [4,27–30]. These studies were conducted in restricted geographical areas, including victims in a single health zone or town. A study conducted at PH between 2004 and 2008 showed an average of 10.4 months’ delay before post-rape consultation in victims of SV. Most were kidnapped by the perpetrator for a long duration [30]. Five reasons were identified: the victim’s expectations of worsening symptoms, poverty, fear of retaliation by family and friends, and stigmatization.

As a center of excellence for the care of victims of SV, PH receives victims from several locations in the DRC, specifically from the eastern region, for specialized medical and surgical care after a sexual assault [31]. Despite the availability of quality care at PH, delayed access to post-rape medical treatment for victims of SV continues to be a major problem in eastern DRC [3,4,32]. Even with an established referral system that integrates the local police, civil society organizations and mobile clinics have not reversed this trend. Therefore, a strategy is required to address this issue. Researchers [2,4,22,23,25] have cited the need to improve timely access to care and identified certain barriers, specifically the need to integrate the patient into a primary healthcare setting [2,22,25]. This requires a multisectoral response to violence against women and SV in particular [25].

Another finding was the victims’ place of residence. SV who lived in the urban areas had a high rate (56%) of consultations in the 72-hour timeframe, which was even higher for those who came from rural areas. In addition, according to Panzi’s holistic care model, victims who had access to information (educated women and girls, and those in contact with local organizations) were able to consult in a timely manner and benefit from care [2]. Regardless of location, Pijlman et al [33], argued that fear and negative social repercussions can prevent victims of sexual violence from seeking medical services and assistance in a timely manner after an assault is exacerbated by feelings of shame, guilt, and embarrassment. Victims may also fear judgment or disbelief, which may discourage them from reporting the violence. A study carried out in rural North Kivu has confirmed the susceptibility of victims to early care-seeking. Referral organizations do not have a major impact on facilitating access to care [14].

In this study, clinical discomfort and fear of pregnancy, were found to influence decisions to seek care. Studies have shown that unwanted pregnancies, reproductive coercion, and the potential consequences of pregnancy complications may contribute to survivors’ hesitancy to access medical services [34]. Fear-related reasons, such as worries about negative pregnancy outcomes, can impact the behaviors and decisions of survivors, including their willingness to seek medical care [35].

Social and cultural factors influence whether a victim seeks medical care within 72 h or at all [27]. This may include fear of rejection by the family or community, social stigmatization, and fear of ineligibility for marriage in the future. Knowledge and physical access barriers are key contributors [22,36,37].

During the study, we observed that SV was mostly perpetrated by civilians with direct or semi-direct relationships with the victim. This can be seen in the eastern part of the DRC, which has experienced war instability over the last three decades and/or where rape was used as a weapon of war [6]. Over time, the growing culture of SV in the community has been underpinned by continued instability, impunity over sexual crimes, and recurring wars. Thus, the profile of the perpetrator gradually changed from armed perpetrators to civilians known or unknown by the victim. Poor demobilization, disarmament, and reintegration can be implicated in an increase in the incidence of rape in the community. Furthermore, the fact that children born of rape and those who witnessed their parents’ rape were not appropriately cared for could explain this situation.

The results of this study allow for the development of a baseline for the fight against SV in the eastern DRC and in other regions with similar environments. This study has implications for improving health policies and practices for preventing SV. In a systematic review measuring the experiences and outcomes of victims who visited a health facility after SV, Caswell et al. [38] suggested a validated and standardized approach considering the victim’s opinion of his or her health condition and experience with the care received. Several strategies should be implemented to ensure early access to care. The holistic PH one-stop care model can be replicated across all health zones throughout the country. This may be integrated with the community health system to bring care closer to victims. In addition to the efforts already made, this advocacy will help ensure access to a post-exposure prophylaxis kit for the early care of victims to avoid consequences.

Emergency care and use of the post-exposure prophylaxis kit remained low for 72 hours. Therefore, efforts should be made to reduce the number of barriers. Intensifying community sensitization, along with promoting education for girls and women, is crucial for addressing social dilemmas. Moreover, the duplication of holistic care model centers or the integration of emergency victims’ assistance into primary health care policies will be fundamental for access to care issues and the prevention of complications. Finally, the improvement of adequate transportation system infrastructure is fundamental to overcoming physical obstacles in obtaining crucial medical care.

One of the strengths of this study is the large sample size and the inclusion of victims of all ages from various cities in eastern and other provinces of DRC. However, this study was limited by its retrospective design. It did not consider victims in the community who were not present at the hospital, or those treated in other settings. This study did not consider care received from other facilities. A larger study should be conducted to assess the national level of access to timely care for SV victims in conflict and post conflict contexts. In addition, research is needed to capture the personal experiences, emotions, or reasons behind delays in seeking care, which are important for a more nuanced understanding of care-seeking behavior.

## Conclusions

Accessing emergency medical care following conflict-related sexual violence remains a challenge in the eastern DRC. Obstacles such as age, location, education level, painful urination as a clinical complaint, and fear of pregnancy were identified.

Several strategies should be implemented to ensure early access to care. Panzi’s holistic care model should be replicated in all health zones of the country by integrating it into the health system to bring care closer to the victims. The efforts already made, advocating early access to post-exposure prophylaxis kits will help to prevent long-term sequelae. In addition, it provides the opportunity, as providers, to educate women on their choices, especially those who do not have access to this information.

## Abbreviations

PEP kit: post-exposure prophylaxis kit
DRC: The Democratic Republic of Congo
SV: sexual violence
PH: Panzl Hospital
